# Active Smoking Induces Aberrations in Digestive Tract Microbiota of Rats

**DOI:** 10.3389/fcimb.2021.737204

**Published:** 2021-11-29

**Authors:** Xiang Wang, Pei Ye, Li Fang, Sheng Ge, Fan Huang, Peter J. Polverini, Weiwei Heng, Lichun Zheng, Qingang Hu, Fuhua Yan, Wenmei Wang

**Affiliations:** ^1^ Nanjing Stomatological Hospital, Medical School of Nanjing University, Nanjing, China; ^2^ Department of Periodontics & Oral Medicine, University of Michigan School of Dentistry, Ann Arbor, MI, United States

**Keywords:** smoking, microbiota, digestive tract, gastrointestinal tract, oral cavity

## Abstract

Cigarette smoking could have certain effects on gut microbiota. Some pioneering studies have investigated effects of active smoking on the microbiome in local segments of the digestive tract, while active smoking-induced microbiome alterations in the whole digestive tract have not been fully investigated. Here, we developed a rat model of active smoking and characterized the effects of active smoking on the microbiota within multiple regions along the digestive tract. Blood glucose and some metabolic factors levels, the microbial diversity and composition, relative abundances of taxa, bacterial network correlations and predictive functional profiles were compared between the control group and active smoking group. We found that active smoking induced hyperglycemia and significant reductions in serum insulin and leptin levels. Active smoking induced region-specific shifts in microbiota structure, composition, network correlation and metabolism function along the digestive tract. Our results demonstrated that active smoking resulted in a reduced abundance of some potentially beneficial genera (i.e. *Clostridium*, *Turicibacter*) and increased abundance of potentially harmful genera (i.e. *Desulfovibrio*, *Bilophila*). Functional prediction suggested that amino acid, lipid, propanoate metabolism function could be impaired and antioxidant activity may be triggered. Active smoking may be an overlooked risk to health through its potential effects on the digestive tract microbiota, which is involved in the cause and severity of an array of chronic diseases.

## Introduction

Cigarette smoking remains the leading cause of disease burden worldwide. Cigarette smoking increases the risk of oral leukoplakia, oral cancer, periodontitis, gastric ulcer, atrophic gastritis, gastric cancer, Crohn’s disease, ulcerative colitis, inflammatory bowel disease, colon cancer, etc.

A large variety of symbiotic microorganisms inhabit the digestive tract and constitute a complex microecosystem, and microbiotas along the digestive tract play a critical role in maintaining host physiological homeostasis (Li et al., 2017; Sedano-Nunez et al., 2018). Some pioneering studies have indicated that smoking can alter microflora composition. At the phylum level, smoking reduced the relative abundance of Firmicutes (Lee et al., 2018; Sublette et al., 2020; Yang et al., 2021) and Bacteroidetes (Stewart et al., 2018), while enhanced that of Cyanobacteria (Huang and Shi, 2019; Sublette et al., 2020; Li et al., 2021c; Yang et al., 2021), Tenericutes (Huang and Shi, 2019), and TM7 (Moon et al., 2015). At the genus level, *Desulfovibrio* (Kato et al., 2010), *Paraprevotella* (Zhang et al., 2020), *Staphylococcus* (Kelley et al., 2021), *Corynebacterium* (Moon et al., 2015; Kelley et al., 2021), and *Xanthomonas* (Huang and Shi, 2019) were enriched in smoking individuals, while *Clostridium* (Wang et al., 2021), *Lactococcus* (Huang and Shi, 2019), *Morganella* (Huang and Shi, 2019) were depleted in smoking individuals.

The digestive tract constitutes several sequential segments and microbiomes in each segment are obviously different due to distinct microenvironments. Therefore, it is necessary to investigate the effects of active smoking on the whole gastrointestinal microbiomes systematically and integrally. Although some studies have indicated that active or passive smoking-induced shifts in microbiomes in a certain segment of the gastrointestinal tract of humans and rodents (Vogtmann et al., 2015; Allais et al., 2016; Wu et al., 2016; Yu et al., 2017; Lee et al., 2018; Shanahan et al., 2018; Stewart et al., 2018; Al-Zyoud et al., 2019; Huang and Shi, 2019; Karabudak et al., 2019; Al Bataineh et al., 2020; Nolan-Kenney et al., 2020; Sublette et al., 2020; Tam et al., 2020; Wirth et al., 2020; Jia et al., 2021; Prakash et al., 2021; Yang et al., 2021; Yoon et al., 2021), the overall effects that active smoking has on the whole digestive tract microbiome are unclear. Therefore, we developed a rat model of active smoking and aimed to determine the effects of active smoking on community structure and bacterial abundance along the digestive tract and on blood glucose and related factor levels.

Our results demonstrated that active smoking reduced the abundance of beneficial genera but increased that of potentially harmful genera. Our findings contribute to understanding smoking-induced microbial dysbiosis in the digestive tract. Some treatments using probiotics, prebiotics, and postbiotics may provide benefits in restoring microecology equilibrium.

## Materials and Methods

### Laboratory Animals and Cigarettes

This study was carried out according to the *National Guide for the Care and Use of Laboratory Animals*. All experimental procedures were approved by the Ethics Committee of Nanjing Stomatological Hospital, Medical School of Nanjing University [IRB Approval Number: 2018NL-008 (KS)] and the Animal Ethical and Welfare Committee of Nanjing University (IACUC-D2102043). Five-week-old Wistar rats, weighing 145–155 g were provided by Vital River Laboratory Animal Technology Co., Ltd (Beijing, China). After arrival at our facilities, each rat was housed in its own cage in a specific pathogen-free facility to avoid microbiota transfer as previously described (Laukens et al., 2016). Rats were housed in cages under controlled ambient temperature (22 ± 3°C) and humidity with a strict 12-h light/12-h dark cycle. Rats were provided with tap water and a standard laboratory chow diet. After one week of acclimatization, the rats were divided into two groups with equivalent average body weights and standard deviations. The rats in the control group (n = 10, male 5 and female 5) were given intraoral delivery of room air. The rats in the active smoking group (n = 9, male 5 and female 4) were given intraoral delivery of cigarette smoke. The rats in the control group and active smoking group were separated in different rooms. Both groups of rats were kept in the animal retainers twice a day (20 min per time in the morning and afternoon) to receive intraoral delivery of room air or cigarette smoke. The total duration of the experiment was 3 months. 3R4F Research Cigarettes (Kentucky Tobacco Research Institute, Lexington, KY, USA) were used in this experiment. Each cigarette contained the following ingredients: tar 9.4 mg, nicotine 0.73 mg, carbon monoxide 12.0 mg.

### Active Smoking Protocol

We developed an intraoral smoking exposure apparatus for rodents that simulates human active smoking. Briefly, the intraoral smoking exposure apparatus included animal retainers, intraoral smoking pipes, cigarette smoke delivery tubes, peristaltic pumps (LongerPumper, Boonton, NJ, USA), and a hood over the smoking rats to evacuate the extra smoke from the environment. During the treatment process, the body of the rats was retained in a plastic hollow cylinder. The delivery tube was connected to the filter tip of a lit cigarette at one end and connected to the intraoral smoking pipe at the other end. The intraoral smoking pipe was sterile and replaced between rats. Then, a peristaltic pump at a speed of 30 rpm was used to automatically draw, deliver and spray cigarette smoke into the oral cavity of rats. Approximately one week after the first smoking treatment, the rats became addicted to active smoking, and it could be observed that the rats smoked with the oral cavity by regular cheek blowing. A video as supplemental information shows that a rat was actively smoking.

### Buccal Swabs, Gastrointestinal Contents and Blood Sampling

After 3 months, microbial samples were collected from the oral cavity by swabbing over the buccal mucosa of rats. The animals were then deeply anesthetized *via* intraperitoneal injection of sodium pentobarbital (2%, 0.2 ml/100g). The abdomen was sterilized with 75% ethanol, and a midline abdominal incision was made. Blood was collected *via* abdominal main vein puncture and centrifuged for further serum biochemical analyses. Then, the rats were sacrificed. Subsequently, the stomach, small intestine, cecum and colon were isolated. The gastrointestinal contents were rapidly collected through a sterile incision in the stomach (gastric antrum), small intestine (middle segment of the jejunum), cecum (body of cecum), and colon (middle segment) while avoiding junction sites of these regions (Li et al., 2017). The serum, swab, and content samples were rapidly collected, flash-frozen with liquid nitrogen, and immediately stored at -80°C until analysis.

### Biochemical Assays

Serum level of cotinine, the primary metabolite of nicotine, was assayed by using liquid chromatography/mass spectrometry. Serum corticosterone level was measured by rat enzyme immunoassay assay kits (Cayman Chemical, Ann Arbor, MI, USA) according to the manufacturer’s instructions. Serum epinephrine level was measured by rat enzyme immunoassay assay kits (Biovision, Milpitas, CA, USA) according to the manufacturer’s instructions. Serum gastrin 1 level was measured by rat enzyme immunoassay assay kits (Abcam, Cambridge, MA, USA) according to the manufacturer’s instructions. Serum glucose level was determined by rat glucose colorimetric assay kits (Cayman Chemical, Ann Arbor, MI, USA) according to the manufacturer’s instructions. Serum GHbA1c level was measured by rat enzyme-linked immune sorbent assay kits (Cusabio, Houston, TX, USA) according to the manufacturer’s instructions. Serum insulin, leptin, and unacylated ghrelin levels were measured by rat enzyme immunoassay assay kits (Cayman Chemical, Ann Arbor, MI, USA) according to the manufacturer’s instructions. Serum adiponectin level was measured by rat enzyme immunoassay assay kits (Abcam, Cambridge, MA, USA) according to the manufacturer’s instructions.

### DNA Extraction and PCR Amplification

DNA extraction was performed as described previously (Clos-Garcia et al., 2019). Bacterial genomic DNA was extracted from the buccal swab and gastrointestinal content samples using a DNA extraction kit (Omega Bio-tek, Norcross, GA, USA) according to the manufacturer’s protocols. Agarose gel electrophoresis was used to assess the DNA integrity and size. DNA concentration and purity were evaluated using a Qubit 2.0 fluorometer (Thermo Fisher Scientific, Waltham, MA, USA). The V4 region of the bacterial 16S ribosomal RNA gene was amplified by PCR using the primers 515 F (GTG CCA GCM GCC GCG GTA A) and 806 R (GGA CTA CHV GGG TWT CTA AT) (Kaakoush et al., 2017).

### Library Construction and Illumina MiSeq Sequencing

The PCR products were separated by gel electrophoresis and purified using an AxyPrep DNA Gel Extraction Kit (Axygen Biosciences, Union City, CA, USA) according to the manufacturer’s instructions. Bacterial 16S rDNA libraries were constructed according to the 16S Metagenomic Sequencing Library Preparation guide from Illumina (Forest City, CA, USA). Only libraries without primer dimers and contaminant bands tested with an Agilent High Sensitivity DNA Kit were used for sequencing *via* Illumina MiSeq. The equimolar purified products were pooled and paired-end sequenced (2 × 250) on an Illumina MiSeq platform according to standard protocols at BGI-Shenzhen (Shenzhen, China). Prior to submission for sequencing, libraries were quality checked. As an added quality control measure, the software package QIIME pipeline was used to filter out and discard poor-quality sequence reads using the default settings (Liu et al., 2018).

### Bioinformatics Analyses

Operational Taxonomic Units (OTUs) based on the 16S rRNA gene sequences were determined using QIIME’s UCLUST algorithm, and taxonomy assignment was performed using the RDP classifier (Bokulich et al., 2018). The relative abundance of each OTU was determined as a proportion of the sum of sequences for each sample.

Alpha diversity analyses estimated from observed species (Sobs), Chao 1, abundance-based coverage estimate (ACE), Shannon, and Simpson indices-based measurements were used to evaluate microbial richness, evenness and community diversity based on the OTUs. The alpha diversity metric was determined using mothur software v.1.31.2, and rarefaction curves were visualized with R program v.3.1.1 (Hall et al., 2017; Lopez-Garcia et al., 2018).

Beta diversity analyses were used to determine the degree of dissimilarity between pairs of bacterial communities using QIIME v.1.80. The unweighted, weighted UniFrac and Bray-Curtis distance matrix, which measures pairwise taxonomic dissimilarity between microbial populations, was analyzed with an unsupervised clustering algorithm (Wu et al., 2018). The beta diversity metric was visualized with a matrix heatmap constructed using the *aheatmap* function from the *NMF* package in R program v.3.1.1 (Gaujoux and Seoighe, 2010).

Taxonomic assignments of the microbiome populations were made according to the OTU annotation within QIIME v.1.80, presenting the taxonomic profiling of the samples at each taxonomic level (Liu et al., 2018).

Heatmaps of bacterial abundance showed the bacterial distribution in five regions along the digestive tract. Log10-transformed relative abundances of bacteria were visualized using the gplots package in R program v.3.1.1 (Li et al., 2020b).

The linear discriminant analysis (LDA) effect size (LEfSe) method was used for statistical analysis at different taxonomic levels (Segata et al., 2011). Cladogram constructed using the LEfSe method to indicate the phylogenetic distribution of active bacteria that were remarkably enriched. LDA scores showed significant bacterial differences within groups at different taxonomic levels.

The common and unique OTUs in different groups were determined and compared using the VennDiagram package in R program v.3.1.1 to generate the Venn diagram (Lam et al., 2016).

Network analysis was conducted to identify correlations between core genera. The inter-generic associations were identified using Spearman’s rho correlation coefficient. Network analyses were visualized in network plots generated by Cytoscape software v.3.5.1 (Xi et al., 2019).

The biological functions of the microbial community were predicted and annotated with KEGG pathways using the PICRUSt algorithm (Li et al., 2017). The OTU contribution to the abundances of functional KEGG pathways was calculated by summing the abundance contribution of each OTU for each KEGG orthology that belonged to the same pathway and then normalizing by the number of samples. Differentially enriched KEGG pathways or modules were identified according to their reporter score (Chen et al., 2017b).

### Statistical Analyses

Most biochemical data and the abundances of the bacteria are presented as the median and interquartile range. Non-parametric statistical methods were used throughout the study. Statistical comparison of two groups was performed with the nonparametric Mann-Whitney *U*-test as described previously (Chu et al., 2017; Clos-Garcia et al., 2019). In each case, *p*-values ≤ 0.05 were considered statistically significant. The analyses were performed with the Statistical Package for the Social Sciences, version 18.0 (SPSS, Chicago, USA).

## Results

### Active Smoking Increased Serum Cotinine Level and Reduced Body Weight Gain

**Figure 1:**
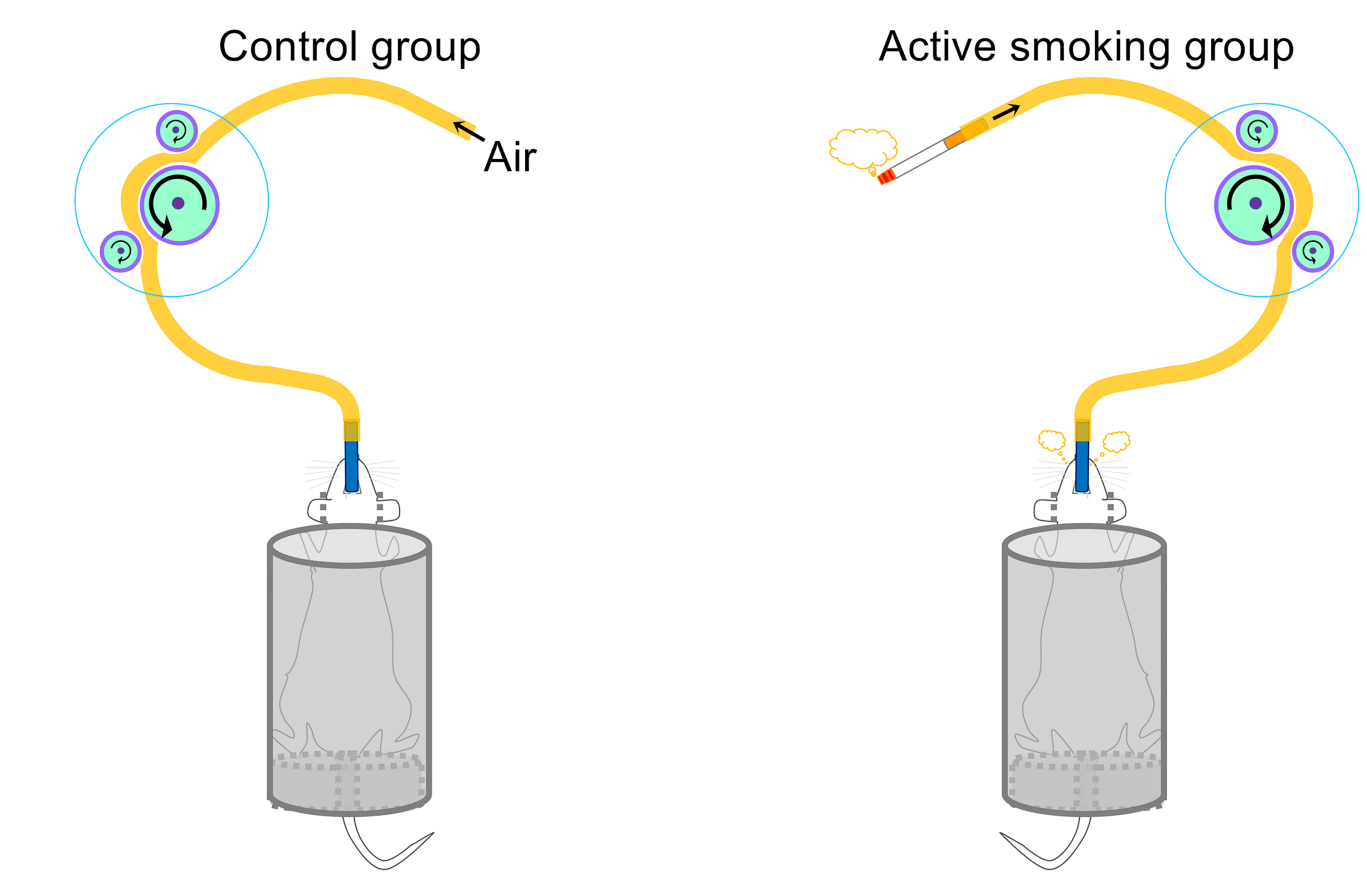
Schematic of the control and active smoking groups.

**Figure 1** shows a schematic diagram of the control and rat model of active smoking using in the present study. The serum cotinine concentration was 2.813 ± 0.944 ng/ml in rats in the active smoking group, while that in the control group rats was zero (*p* < 0.001) (**Table 1**). There was no statistically significant differences in serum gastrin 1, corticosterone and epinephrine levels between the control group and active smoking group (**Table 1**). Our results showed that rats in the active smoking group gained less weight compared with rats in the control group (*p* < 0.01) (**Supplementary Figure S1**).

**Table 1:** Comparison of serum parameters concentration between the control group and active smoking group.

Parameters	Control group		Active smoking group		*p*-value
	Median	IQR	Median	IQR	
Cotinine (ng/ml)	0	0-0	2.83	2.28-3.54	**<0.001** ^***^
Glucose (mg/dl)	94.46	73.02-97.35	113.00	103.73-138.49	**0.002** ^**^
GHbA1c (ng/ml)	40.38	31.24-50.75	44.97	34.42-47.78	0.967
Insulin (ng/ml)	34.02	27.30-37.21	23.52	20.82-24.89	**0.006** ^**^
Leptin (pg/ml)	2955.23	2513.22-3614.14	2060.54	971.95-2179.53	**0.011** ^*^
Adiponectin (pg/ml)	6781.37	2460.91-13764.70	4227.80	3121.15-4701.30	0.624
Ghrelin (pg/ml)	34.02	27.30-37.21	76.83	22.88-467.08	0.935
Gastrin 1 (pg/ml)	378.33	187.28-591.37	254.66	206.07-536.91	0.838
Corticosterone (pg/ml)	1526.81	829.71-2301.59	1617.62	1356.82-2812.03	0.514
Epinephrine (pg/ml)	2347.72	1313.69-4583.56	2811.60	1342.96-4280.89	0.806

IDR: interquartile range; GHbA1c: glycosylated hemoglobin A1c; *: p < 0.05 versus control group, **p < 0.01 versus control group, ***p < 0.001 versus control group, Bold values: statistically significant differences.

### Active Smoking Induced Hyperglycemia and Shifts in Related Factor Levels

Biochemical analyses indicated that the serum glucose level in the active smoking group was significantly higher than that in the control group (*p* < 0.01). Moreover, the serum insulin and leptin levels in the active smoking group were remarkably lower than those in the control group (*p* < 0.01 and *p* < 0.05, respectively). However, the serum glycosylated hemoglobin A1c (GHbA1c), adiponectin and ghrelin levels were not significantly altered in the active smoking group compared with the control group (*p* > 0.05) (**Table 1**).

### High-Throughput Sequencing Data

**Figure 2:**
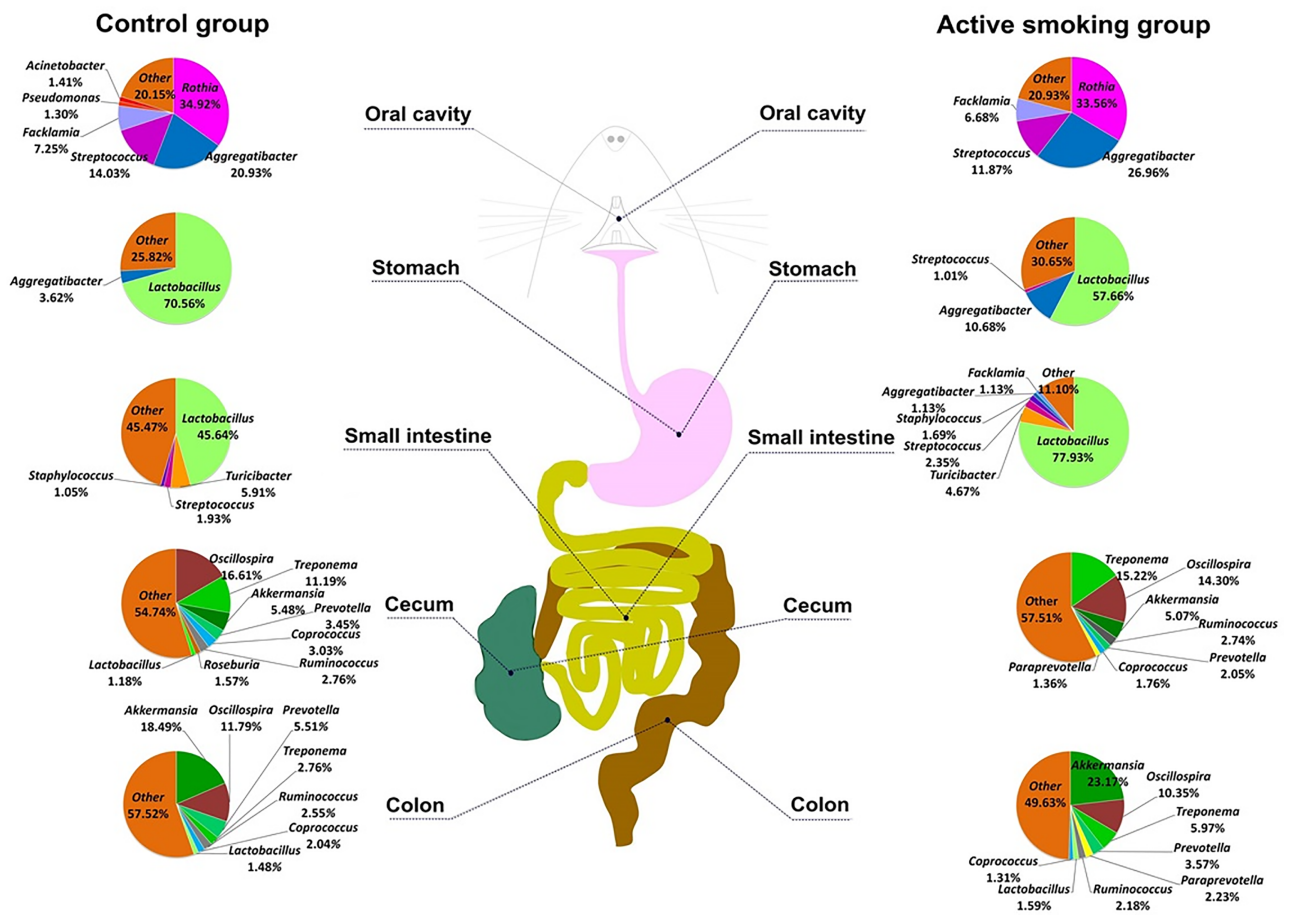
The schematic of the digestive tract of rats and constituents of the microbiome in the control and active smoking groups. Pie charts for the bacterial genera at five regions along the digestive tract according to the median relative abundance. Each genus is indicated by different colors. Genera with a relative abundance lower than 1% are labeled together as ‘others’ (orange).

Except for two colonic content samples that were unavailable in a rat in the control group and a rat in the active smoking group, all 93 specimens met the requirements for library establishment. In total, 4,245,900 sequence reads with a mean length of 252 bp were obtained from all the samples (the oral swabs and gastric, small intestinal, cecal, and colonic contents) in the control and active smoking groups. Each sample was covered by an average of 45,655 reads, arrange from 36,789 to 46,773. **Figure 2** shows a schematic of five regions along the digestive tract and the microbiome constituents in each region in the control and active smoking groups.

### Effects of Active Smoking on Community Structure of the Digestive Tract

**Figure 3:**
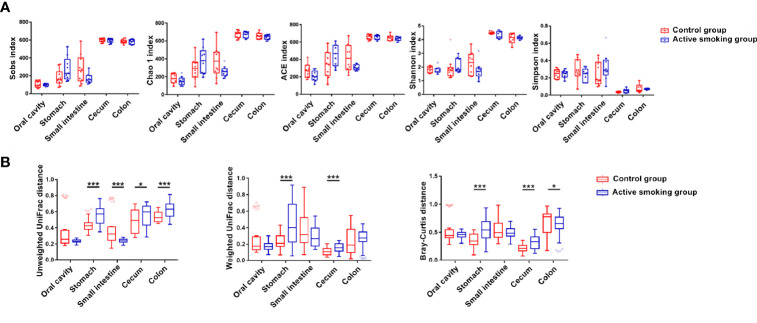
Bacterial community structure along the digestive tract of rats in the active smoking group compared with the control group. **(A)** Box plots of alpha diversity measured by five indices. Boxes denote the interquartile range (IQR) between the first and third quartiles, and the line inside the boxes denote the median. Circles represent data points beyond the whiskers. **(B)** Pairwise comparison of median unweighted, weighted UniFrac and Bray-Curtis distances between the active smoking group and control group. * and *** denote *p* < 0.05 and *p* < 0.001, respectively.

Regardless of the smoking status, bacterial species richness and diversity were significantly different between most regions along the digestive tract. The cecal microbiota had the greatest diversity (596 unique OTUs per sample on average), followed by the colonic microbiota (582 unique OTUs per sample on average), and the oral microbiota had the lowest diversity (102 unique OTUs on average). The gastric and small intestinal microbiota shared the same diversity (both with 225 unique OTUs on average). Our analyses indicated that alpha diversity was generally increased along the digestive tract. The stomach and small intestine harbored a more diverse microbial community than the oral cavity, while the cecum and colon possessed a more diverse microbial community than the stomach and small intestine (**Figure 3A**).

The alpha diversity in each region did not remarkably differ between the control group and active smoking group (*p* > 0.05) (**Figure 3A**). Based on analyses of the unweighted UniFrac distance metrics, the bacterial communities in the gastric, small intestinal, cecal or colonic contents in the control and active smoking groups were markedly different in beta diversity (*p* < 0.001, *p* < 0.001, *p* < 0.05, and *p* < 0.001, respectively) (**Figure 3B**). Based on analyses of the weighted UniFrac distance metrics, the bacterial communities in the gastric or cecal contents in the control and active smoking groups were significantly different in beta diversity (*p* < 0.001 and *p* < 0.001, respectively) (**Figure 3B**). Based on the Bray-Curtis distance metrics, the bacterial communities in the gastric, cecal or colonic contents in the control and active smoking groups were also clearly distinct by beta diversity (*p* < 0.001, *p* < 0.001, and *p* < 0.05, respectively) (**Figure 3B**).

**Figure 4:**
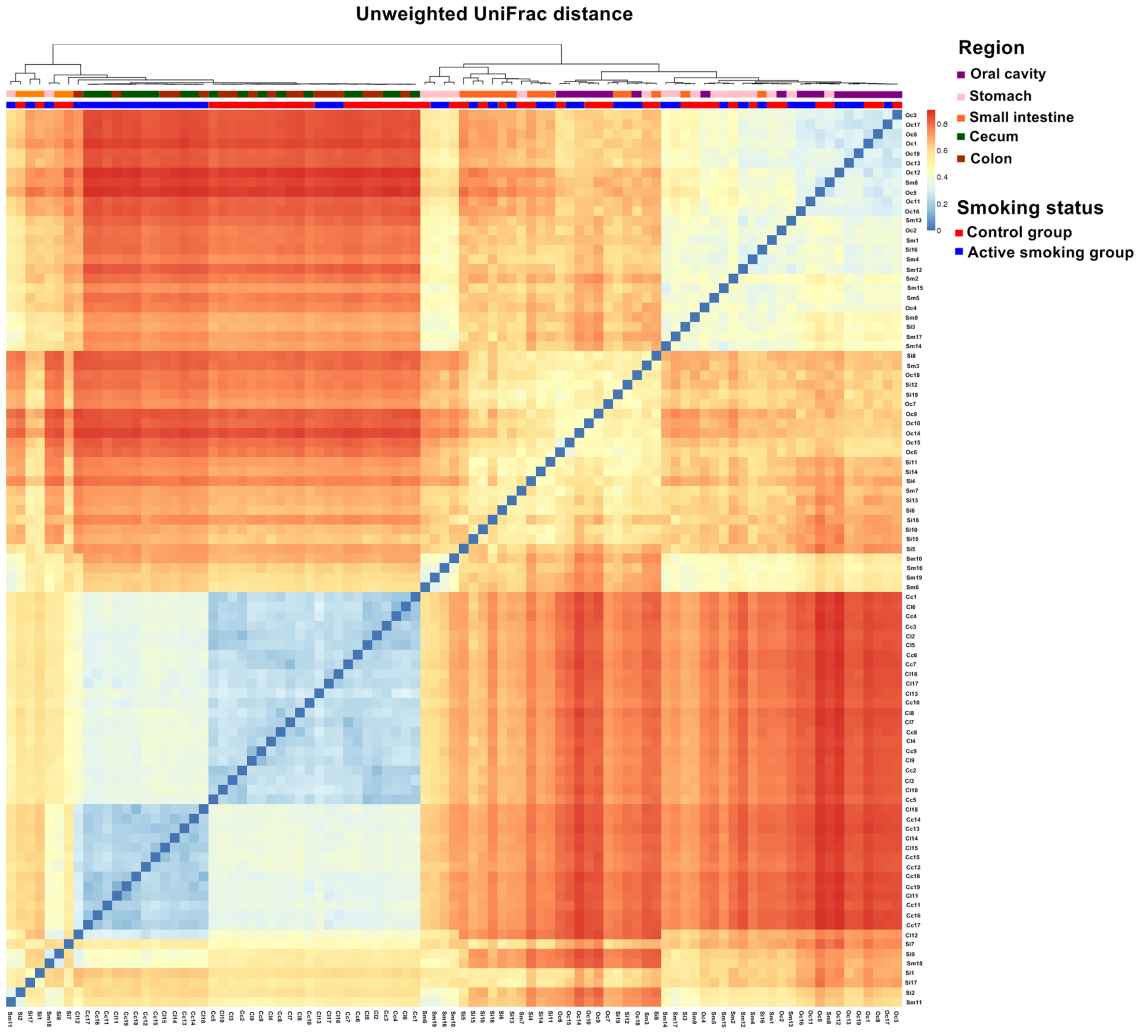
Matrix heatmap based on phylogenetic unweighted UniFrac distance displays the overall community structure differences along the digestive tract according to active smoking status. Distinctive clustering in the control group and active smoking group is visualized in the cecal and colonic bacterial communities.

Our analyses indicated that the community structure of the stomach was analogous to that of the small intestine, while the cecum and colon shared a similar microbial community (**Figure 4** and **Supplementary Figures S2, S3**). Moreover, the community structure of the oral cavity was closer to that of the stomach and small intestine in the unweighted, weighted UniFrac or Bray-Curtis distance (**Figure 4** and **Supplementary Figures S2, S3**). A matrix heatmap shows distinctive clustering of the cecal and colonic bacterial communities by active smoking status based on the unweighted UniFrac distance analyses (**Figure 4**). A matrix heatmap shows distinctive clustering of the oral cavity, gastric and small intesinal bacterial communities by active smoking status based on the Bray-Curtis distance analyses (**Supplementary Figure S2**). A matrix heatmap shows relative weak clustering of the digestive tract bacterial communities by active smoking status based on the weighted UniFrac distance analyses (**Supplementary Figure S3**).

**Figure 5:**
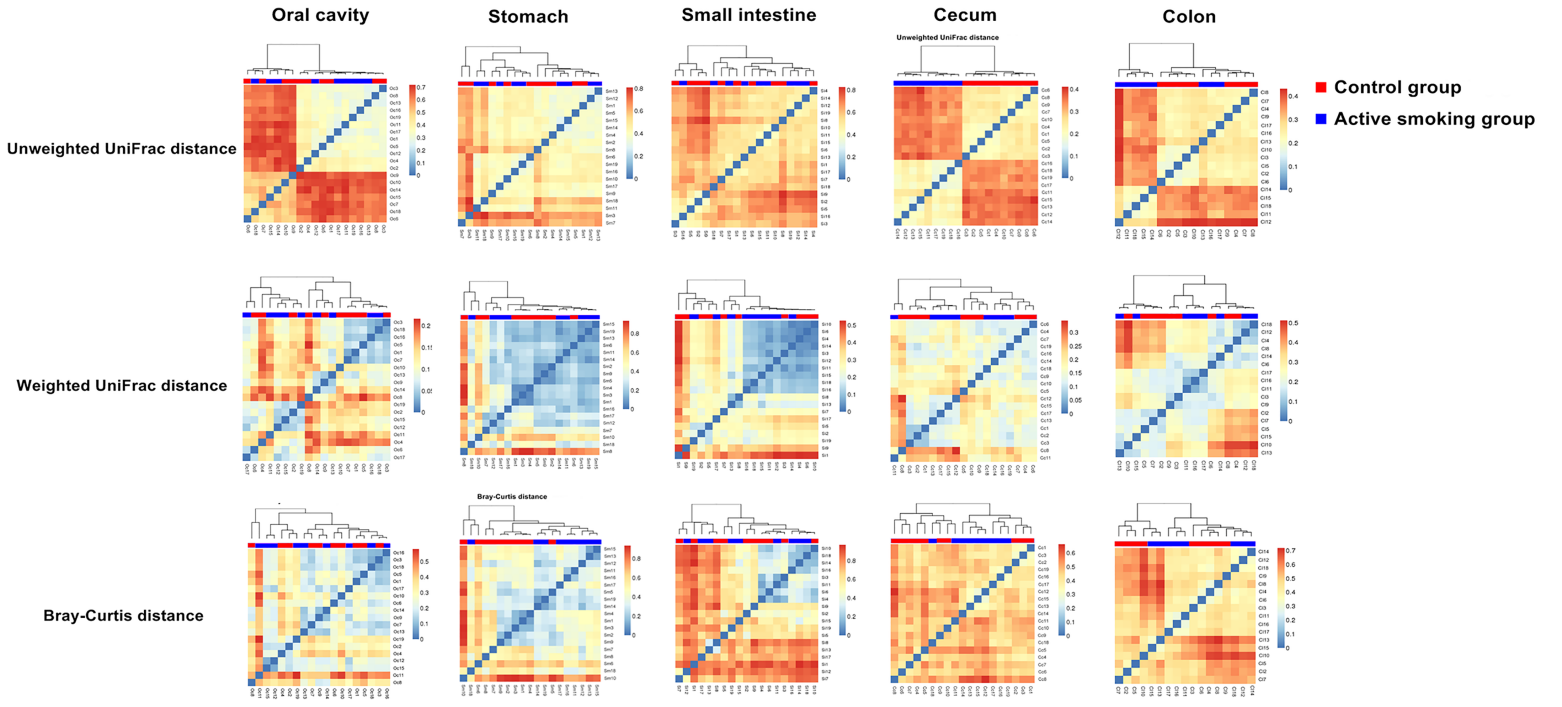
Matrix heatmaps based on beta analysis metrics separately show the community structure dissimilarity in the oral cavity, stomach, small intestine, cecum, and colon according to active smoking status.

Matrix heatmaps based on the unweighted UniFrac analyses show distinctive clustering in the control group and active smoking group in the oral cavity, cecal and colonic bacterial communities (**Figure 5**). Matrix heatmaps based on the Bray-Curtis and weighted UniFrac analyses show distinctive clustering in the control group and active smoking group in the gastric, cecal and colonic bacterial communities (**Figure 5**).

### Community Composition of the Digestive Tract Modified by Active Smoking

**Figure 6:**
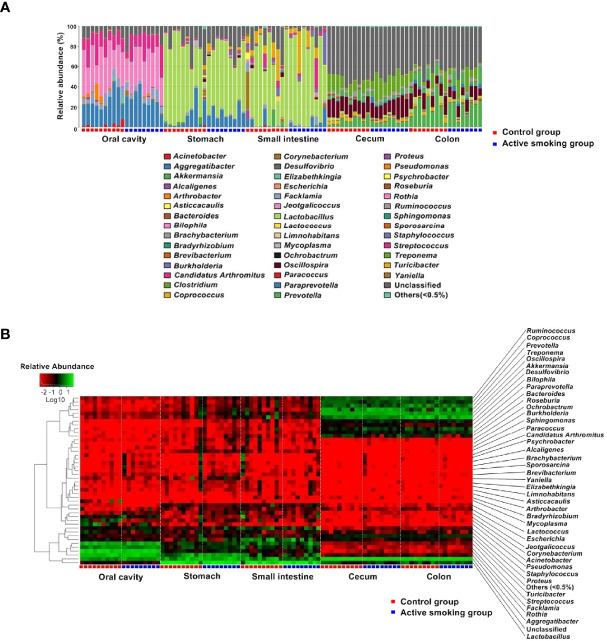
Comparison of the microbiotal composition along the rat digestive tract between the control group and active smoking group. **(A)** Distribution of bacterial genera and their relative abundance among microbiota in different samples are shown in a bar plot. **(B)** Heatmap showing log10-transformed relative abundances of bacteria in different samples at the genus level.

From the phylum to the species level, the taxonomic analysis revealed the enriched taxa in the control group or active smoking group (**Supplementary Tables S1-S5**). Not surprisingly, both the relative abundance heatmap and taxonomic profiling bars showed that the overall microbial composition significantly varied along the digestive tract (**Figure 6**). Despite this, Firmicutes was the most abundant phylum in the gastrointestinal regions.

At the phylum level, rats in the active smoking group possessed significantly lower relative abundances of Bacteroidetes (*p* < 0.01) and higher relative abundances of Cyanobacteria (*p* < 0.05) in the oral cavity. When comparing the composition of the gastric and small intestinal microbiota between the groups, there were no differences in abundance at the phylum level. Rats in the active smoking group bore significantly lower relative abundances of Firmicutes (*p* < 0.05) in the cecum. Rats in the active smoking group possessed significantly higher relative abundances of Tenericutes and TM7 (*p* < 0.05 and *p* < 0.05, respectively) in the colon (**Supplementary Tables S1–S5**).

Overall, at the genus level, the results suggested that active smoking significantly altered the enrichment of low-abundance genera (**Supplementary Tables S1–S5**). Genera with relative abundance higher than 5% seemed to not be directly affected by active smoking. Interestingly, our results showed that the proportions of the class Clostridia, the order Clostridiales and the order Turicibacterales, the family Clostridiaceae and the family Turicibacteraceae, the genus *Clostridium* and the genus *Turicibacter*, and the species *Clostridium perfringe*n*s* were reduced in the active smoking group compared with the control group (**Supplementary Tables S1–S5**).

**Figure 7:**
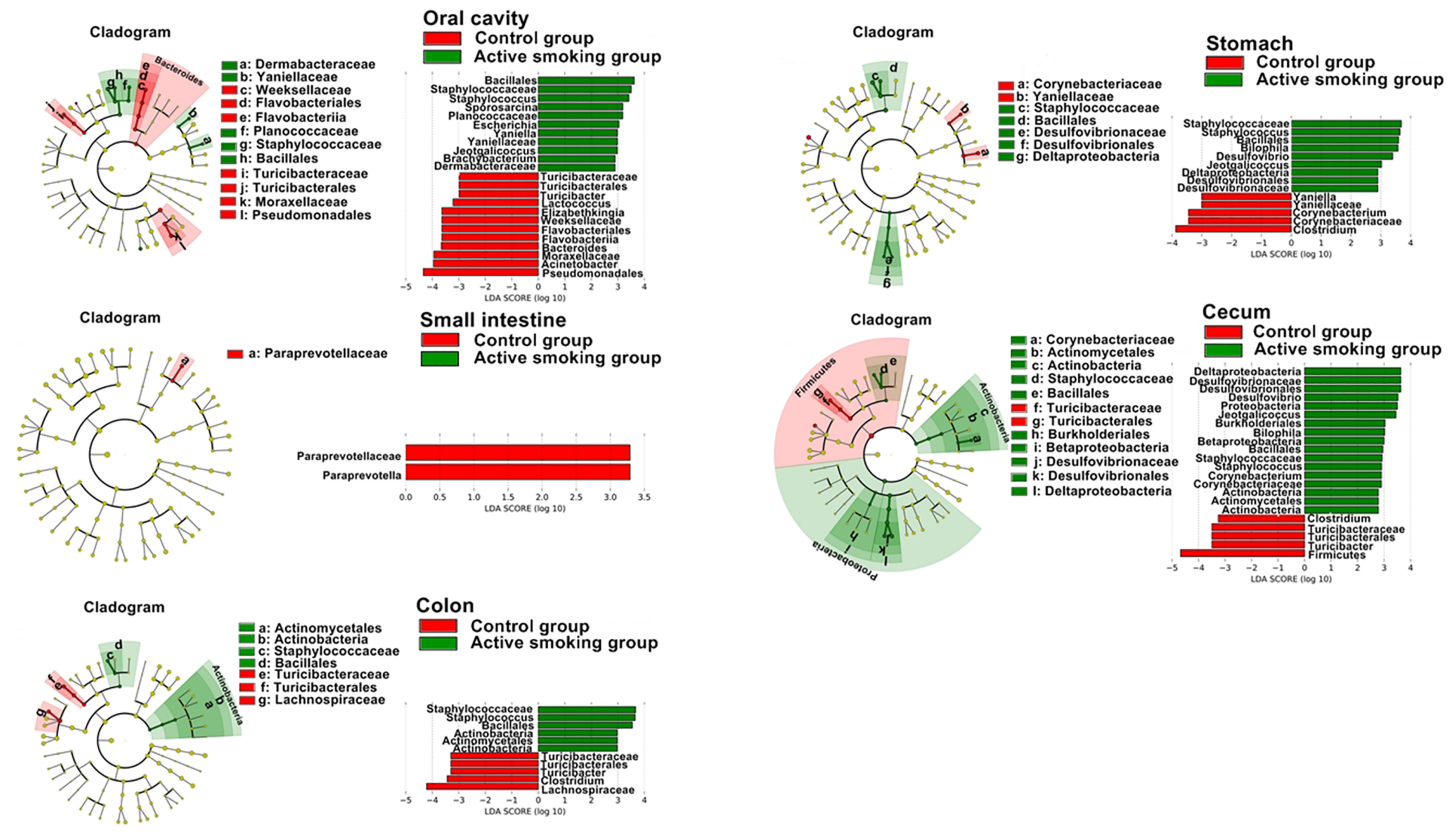
Distinct active taxa identified in the oral cavity, stomach, small intestine, cecum, and colon according to smoking status using LEfSe analysis. Counts were analyzed using LEfSe to identify significant differences in bacterial abundance between the control group and active smoking group. Cladogram constructed using the LEfSe method to indicate the phylogenetic distribution of active bacteria that were remarkably enriched. LDA (Linear Discriminant Analysis) scores showed significant bacterial differences within groups at different taxonomic levels. Red represents the enriched taxa in the control group and green represents the enriched taxa in the active smoking group.

In the class level, the relative abundance of Clostridia was lowered in the oral, small intestinal and cecal samples from the active smoking group (*p* < 0.05, *p* < 0.05 and *p* = 0.05, respectively) (**Supplementary Tables S1, S3, S4**). In the order level, the relative abundance of Clostridiales was decreased in the oral, small intestinal and cecal samples from the active smoking group (*p* < 0.05, *p* < 0.05 and *p* = 0.05, respectively) (**Supplementary Tables S1, S3** and **S4**). At the family level, the relative abundance of Clostridiaceae was decreased in the cecal samples from the active smoking group (*p* < 0.05) (**Supplementary Table S4**). At the genus level, the relative abundance of *Clostridium* was reduced in the gastric and cecal samples from the active smoking group (*p* < 0.05 and *p* < 0.01, respectively) (**Figure 7** and **Supplementary Tables S2, S4**). At the species level, the relative abundance of *Clostridium perfringe*n*s* was lower in the gastric, cecal and colonic samples from the active smoking group (*p* < 0.05, *p* < 0.05, and *p* < 0.01, respectively) (**Supplementary Tables S2, S4, S5**).

In the order level, the relative abundance of Turicibacterales was decreased in the oral, cecal and colonic samples from the active smoking group (*p* < 0.05, *p* < 0.05 and *p* < 0.05, respectively) (**Figure 7**, **Supplementary Tables S1, S4, S5**). At the family level, the relative abundance of Turicibacteraceae was decreased in the oral, cecal and colonic samples from the active smoking group (*p* < 0.05, *p* < 0.05 and *p* < 0.05, respectively) (**Figure 7** and **Supplementary Tables S1, S4, S5**). At the genus level, the relative abundance of *Turicibacter* was reduced in the oral, cecal and colonic samples from the active smoking group (*p* < 0.05, *p* < 0.05 and *p* < 0.05, respectively) (**Figure 7** and **Supplementary Tables S1, S4, S5**).

Besides reduced abundance of potentially beneficial genera (*Clostridium* and *Turicibacter*), our results also demonstrated a clear enrichment in sulfate-reducing bacteria (*Desulfovibrio* and *Bilophila*) and some opportunistic pathogens (*Staphylococcus*, *Jeotgalicoccus*, and *Odoribacter*). At the genus level, the relative abundance of *Desulfovibrio* was increased in the gastric and cecal samples from the active smoking group (*p* < 0.01 and *p* < 0.05, respectively) (**Supplementary Tables S2, S4**). Also, the relative abundance of *Bilophila* was increased in the gastric and cecal samples from the active smoking group (*p* < 0.05 and *p* < 0.05, respectively) (**Supplementary Tables S2, S4**). The genus *Staphylococcus* abundance was increased in the oral, cecal and colonic samples from the active smoking group (*p* < 0.01, *p* < 0.01 and *p* < 0.01, respectively) (**Supplementary Tables S1, S4, S5**). Also, the genus *Jeotgalicoccus* abundance was increased in the oral and cecal samples from the active smoking group (*p* < 0.01 and *p* < 0.01, respectively) (**Supplementary Tables S1, S4**). The genus *Odoribacter* abundance was increased in the cecal and colonic from the active smoking group (*p* < 0.001 and *p* < 0.01, respectively) (**Supplementary Tables S4, S5**).

### Active Smoking Induced Differences in Community Structure and Phylum Abundance Between the Stomach and Small Intestine

**Figure 8:**
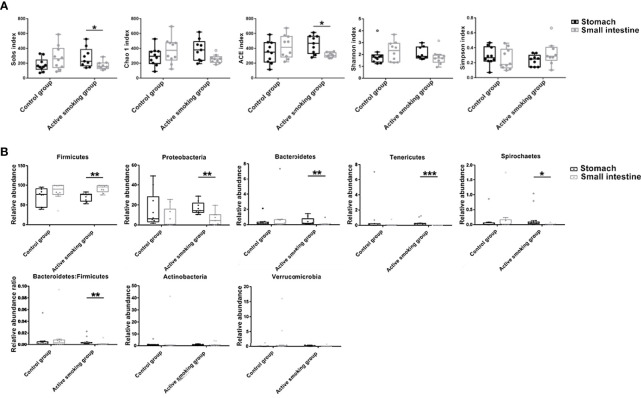
Active smoking induced-differences in bacterial community structure and relative abundance between the stomach and small intestine. **(A)** Relative to the similarity in alpha diversity between two regions in the control group, significantly differential median Sobs and ACE were measured between the stomach and small intestine in the active smoking group. **(B)** Relative to the comparable relative abundances of bacterial phyla between the stomach and small intestine in the control group, significantly distinct relative abundances of bacterial phyla were determined in the active smoking group. *, ** and *** denote *p* < 0.05, *p* < 0.01 and *p* < 0.001, respectively.

Surprisingly, the alpha diversity of the gastric microbiome was significantly higher than that of the small intestinal microbiome in the active smoking group (observed species and ACE metric) (*p* < 0.05), while those of the gastric and small intestinal microbiome in the control group were comparable (*p* > 0.05) (**Figure 8A**). Intriguingly, in the control group, no significant differences in the relative abundance of the Firmicutes, Bacteroidetes, Proteobacteria, Spirochaetes, and Tenericutes phyla were observed between the gastric contents and the small intestinal contents (*p* > 0.05). However, in the active smoking group, the relative abundances of these phyla were significantly distinct between the gastric contents and the small intestinal contents (*p* < 0.05) (**Figure 8B**). At the OTU level, fewer unique OTU-annotated bacterial taxa resided in the oral cavity, small intestine, and colon of the active smoking group compared with the control group. In contrast, more unique OTU-annotated bacterial taxa inhabited the stomach and cecum of the active smoking group compared with the control group (**Supplemental Figure S4**).

**Figure 9:**
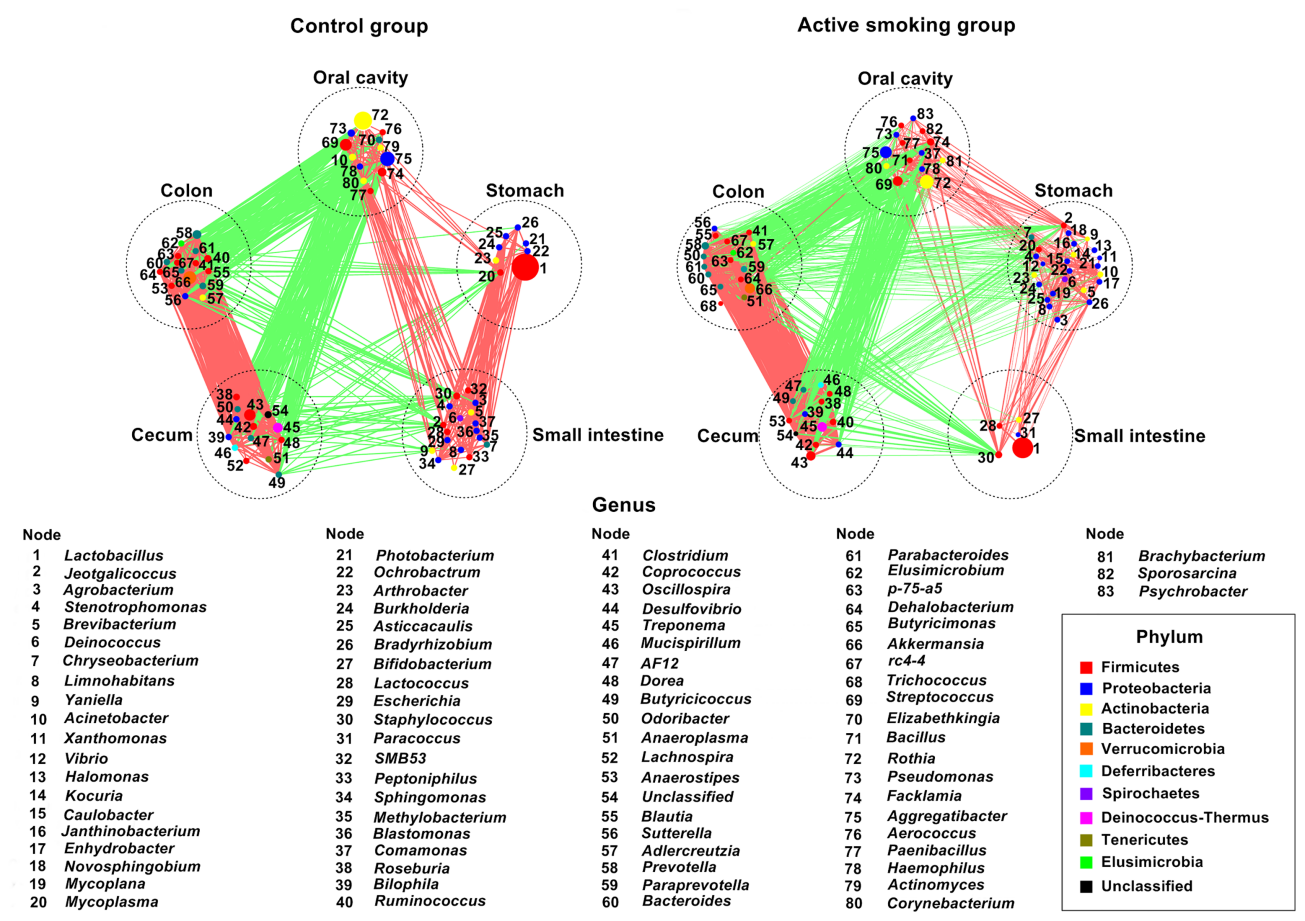
Network plots highlight correlations between positive and negative responders at five regions along the digestive tract of rats in the control group and the active smoking group, arranged in the same order. Prominent aberrations in bacterial community structure in the stomach and small intestine were determined in the active smoking group. The node size is proportional to the mean relative abundance of genera in the enriched population. Edges between nodes represent correlations between the connected nodes, with line width indicating the correlation magnitude. Only lines corresponding to correlations with magnitudes > 0.4 or < -0.4 are shown. Green edges, negative correlations; red edges, positive correlations.

In agreement with the taxonomic profiling and alpha diversity findings, the network analyses revealed prominent shifts in microbial community structure and altered relationships between the gastric and small intestinal flora (**Figure 9**). It was also noted that robust inter-generic networks were translocated from the small intestine flora in the control group into the stomach flora in the active smoking group. Moreover, in contrast with the control group, the core genera of the stomach were significantly enriched while the core genera of the small intestine were profoundly depleted in the active smoking group. Although *Lactobacillus* was the keystone species of the stomach and small intestine in the control group and the active smoking group, its role was significantly reduced within the microbial associations in the stomach and conspicuously enhanced within the microbial associations in the small intestine in the active smoking group.

### Active Smoking Contributes to Altered Microbial Functions Associated With Metabolism

**Figure 10:**
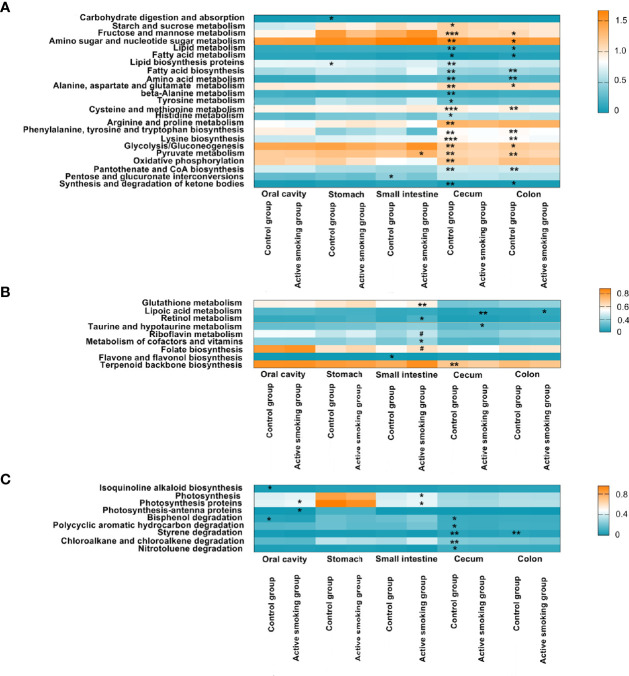
Associations between active smoking status and inferred bacterial functions. Bacterial KEGG pathways significantly enriched in the control group or active smoking group were plotted as heatmaps by region along the digestive tract. Regions of the digestive tract and active smoking status are indicated at the bottom. Each bar represents the median relative abundance of a certain functional KEGG pathway of samples from each digestive tract region. The colors denote the variation in median relative abundance. Blue denotes the lowest abundance, while orange represents the highest abundance. The transition between high and low relative abundance is expressed in white. **(A)** Metabolism or biosynthesis of the primary nutrition substance-related pathways. **(B)** Metabolism or biosynthesis of antioxidant- and vitamin-associated pathways. **(C)** Diabetes-improving chemical biosynthesis-, photosynthesis-, and xenobiotic degradation-related pathways. ^#^denotes *p*-value = 0.05 and ^*^, ^**^ and ^***^ denote *p*-value < 0.05, *p*-value < 0.01 and *p*-value < 0.001 *versus* the control group or active smoking group, respectively.

Functional prediction analyses (**Figure 10**) suggested that microbiota associated with pathways for carbohydrate, lipid, fatty acid, and amino acid metabolism and lipid or amino acid biosynthesis was more pronounced in rats in the control group (*p* < 0.05). However, microbiota associated with metabolism pathways for some antioxidants and vitamins, such as glutathione, lipoic acid, retinol, taurine, hypotaurine, riboflavin, and cofactors, was enriched in the active smoking group (*p* < 0.05).

## Discussion

Although the oral and fecal microbiomes have been well studied, it is challenging to collect gastric and intestinal contents to observe human microbiota shifts in the digestive tract. An animal model is a good alternative to assess the effects of active smoking on the human microbiome along the digestive tract systematically and comprehensively. In the present study, our results indicated that active smoking directly affectd the digestive tract microbiota which then resulted to the alteration of some biochemical components. Chronic cigarette smoke exposure has been reported to modulate cecal microbiota, increase inflammation of the small intestine, and decrease blood leptin level in mice (Dubois-Deruy et al., 2020). A recent study indicated smoking induced gut microbiota dysbiosis, broke metabolism homeostasis and caused insulin resistance in mice (Yang et al., 2021). Another study indicated that smoking modulated the microbial composition of the oral cavity and reduced salivary levels of insulin and leptin (Rodriguez-Rabassa et al., 2018).

Some rodent or human-based studies have confirmed that smoking could result in alterations in community structure and taxonomic abundances in a certain segment of the digestive tract (Vogtmann et al., 2015; Lee et al., 2018; Shanahan et al., 2018; Al-Zyoud et al., 2019; Sublette et al., 2020; Tam et al., 2020; Young et al., 2020; Prakash et al., 2021). Our results further confirmed that active smoking could induce a comprehensive effect on community structures and taxonomic abundances in the whole digestive tract. The present findings showed that beta diversity of the gastrointestinal microbiota was clearly altered by active smoking. These results suggest that smoking can induce a shift in community structure or bacterial profile in the digestive tract.

In the present study, the rats in the active smoking groups had smaller proportional abundances of Clostridia, Clostridiales, Turicibacterales, Clostridiaceae, Turicibacteraceae, *Clostridium*, and *Turicibacter* in the digestive tract than the control rats. Accordingly, the data of our PICRUSt analysis showed that active smoking reduced the microbial community functions, especially in the metabolism of amino acids (alanine, aspartate, glutamate, cysteine, methionine, arginine, proline, beta alanine, and histidine), carbohydrates (starch, sucrose, fructose, mannose, amino sugar, nucleotide sugar, pyruvate, glycolysis, gluconeogenesis), and one of short-chain fatty acids (SCFAs), i.e. propanoate.

*Clostridia* are the most common fermenters of amino acids found in the gut (Young et al., 2020). A recent study revealed that depletion of some Clostridiales was associated with dysfunction in amino acid metabolism and carbohydrate metabolism (Li et al., 2020a). *Clostridium*, as the core gut microbial genus, is closely related to carbohydrate metabolism. There is exact evidence that *Clostridium* acts as the predominant amino acids-fermenting microbe along the digestive tract of humans and animals (Dai et al., 2011). Not surprisingly, consistent with the previous studies, our results suggested that active smoking decreased body weight gain (Ypsilantis et al., 2013; Tong et al., 2020). The present results indicate that active smoking not only induced aberrations in the oral and gastrointestinal microbiomes, but it also led to hyperglycemia, reduced serum insulin and leptin levels. Apart from decreased appetite and changes in serum leptin levels, active smoking also interferes negatively with the gut homeostasis by promoting mucosal inflammation and impairing mucosal barrier integrity (Tam et al., 2020). Therefore, impaired community functions may dampen metabolic function and nutrition absorption, further lowering the body weight gain. Further, our results suggested that active smoking may induce abnormal amino acid and lipid metabolism by reducing abundance of *Clostridium* and *Turicibacter*. *Turicibacter* is a genus in the Firmicutes phylum of bacteria that has most commonly been found in the guts of animals. It was reported that *Turicibacter* has been strongly associated with abnormal lipid and carbohydrate metabolism (Horie et al., 2017; Li et al., 2021a).

In our study, amino acid metabolism and lipid metabolism were significantly decreased in the active smoking group compared that in the control group. Our results demonstrated that active smoking significantly decreased abundances of Turicibacterales, Turicibacteraceae, and *Turicibacter* in the oral, cecal and colonic contents. The decrease we observed here is likely related to the perturbing effects of active smoking on lipid metabolism. Notably, the genus *Turicibacter* is beneficial and anti-inflammatory and plays a protective role in animal models of inflammatory bowel disease (Werner et al., 2011; Song et al., 2017).

In our study, active smoking increased the abundances of *Desulfovibrio* and *Bilophila* in the gastric and cecal contents. As lipopolysaccharide-producing, mucosa-damaging, and pro-inflammatory bacterial communities, *Desulfovibrio* and *Bilophila* have been known as harmful genera (Song et al., 2017). The genera *Desulfovibrio* and *Bilophila* are likely participate in the development of colorectal cancer by producing hydrogen sulfide and promoting chronic inflammation. On one hand, it reduces sulfate to sulfide which has a toxic effect on intestinal epithelial cells and induces abnormal proliferation and metabolism of epithelial cells. On the other hand, it also destroys the intestinal barrier function by inhibiting the oxidation of SCFAs (Chen et al., 2019).

Gut microbiota influence metabolism and immune response mainly by its fermentation products i.e. SCFAs, which regulate glucose and lipid metabolism. SCFAs work as a mediator between gut microbiota and pancreas and improve glucose homeostasis and insulin sensitivity (Mandaliya and Seshadri, 2019). These SCFAs are potent preventive agents against colorectal cancer and inflammation. *Clostridium*, *Turicibacter*, and *Coprococcus* are main SCFAs-producing bacteria, which regulated the gut barrier and immune response as well as the endocrine system (Li et al., 2019). Propionate is a major fermentation product and one of SCFAs in the gut with several health benefits toward energy homeostasis. In case of diseases where microbial dysbiosis is apparent, gut microbial production of propionate may be decreased. Propionate stimulates satiety-inducing hormones, leading to lower energy intake and reducing weight gain and associated risk factors (El Hage et al., 2019). Our analyses indicated that the abundances of *Clostridium*, *Turicibacter*, and *Coprococcus* were significantly decreased by active smoking. Moreover, our results of PICRUSt analyses showed that active smoking reduced propionate metabolism in the cecal and colonic contents. In our study, the abundance of *Desulfovibrio* increased and propionate metabolism decreased in the active smoking group. Sulfate reducing bacteria including the *Desulfovibrio* genera are able to grow on propionate (Sedano-Nunez et al., 2018; Ozuolmez et al., 2020) and sulfate reduction then proceeds with propionate (but not butyrate) as the electron donor (Chen et al., 2017a; He et al., 2019).

Previous studies have determined that long-term smoking can result in a chronic inflammation microenvironment and oxidative stress in the gastrointestinal tract. Active smoking-induced inflammation, oxidative stress and biochemical shifts could lead to structural and functional dysbiosis of the oral and gastrointestinal flora (Papoutsopoulou et al., 2020; Genua et al., 2021). The present results suggest that active smoking can impair nutritional metabolism function and induce activated antioxidant activity. Antioxidant activity in microbial communities in the digestive tract might be reactively triggered by smoking-induced oxidative stress and contribute to alleviation of inflammation.

Some treatments using probiotics, prebiotics, and postbiotics may provide benefits in smoking-induced dysbiosis and disorders. Constructing metabolic networks of a dysbiosis can improve the process of choosing the best treatment. Metabolic networks provide a useful tool to investigate the effectiveness of probiotic treatment (Jansma and El Aidy, 2021). The metabolic products of the abundant microbiota mostly affects the establishment of probiotic bacteria, can be supported with the use of prebiotics. Gut microbiota can be altered/modified using probiotics, prebiotics, synbiotics, and postbiotics such as (SCFAs), and all of these can contribute positively to host health (Adithya et al., 2021). Some prebiotics such as dietary polyphenols exert antimicrobial activities against pathogenic gut microbiota, improve gut metabolism and immunity, impart anti-inflammatory properties, and also provide benefits in various gastrointestinal, metabolic, and neuropsychological disorders (Adithya et al., 2021). Interestingly, a recent study indicated that arabinoxylan can increase the relative abundance of *Clostridium*, while decrease the relative abundance of *Desulfovibrio* and *Bilophila* (Li et al., 2021b).

Our study has several limitations. Firstly, the sample number in each group is small in our study, although the overall sample number is relative large to cover all the segments of the digestive tract. It is necessary to increase sample size in further study. Secondly, the analyses of metabolites in the digestive tract are absent in our study, which would be helpful to strengthen our assessment. Some other biochemical components present in the digestive tract, such as vasoactive intestinal peptide (VIP), somatostatin (SST), and 5-hydroxytryptamine (5-HT), which can reflect the status of the gastrointestinal functions, should be included in further study. Finally, we compared the active smoking group with the control group regardless of gender and other characteristics that could have influenced the microbiomes. In further study, we will use only one sex type (males are preferred since female rats’ hormonal changes are more elaborate which can alter the activities of the body affecting the biochemical activities). Therefore, our results should be interpreted carefully. Further studies are needed to investigate the effects of active or passive smoking on microbiomes, metabonomics, even metagenomics based on large-scale data.

Collectively, our results demonstrated that active smoking altered the gut microbiota of rats through decreasing the abundance of potentially beneficial genera (e.g., *Clostridium*, *Turicibacter*), and increasing the abundance of potentially harmful genera (e.g., *Desulfovibrio*, *Bilophila*). Given that the importance of microecological equilibrium, our findings may be value in understanding how microbial dysbiosis in the gastrointestinal tract is involved in the pathogenesis of smoking-related diseases. Further in-depth studies investigating the animal and human microbiome in the digestive tract will be necessary to gain insight into the pathogenesis of some smoking-related diseases, uncover the intricate and intrinsic underlying mechanism, and identify microbial signatures to pave the way for development of customized therapeutics, probiotics, prebiotics and postbiotics.

## Data Availability Statement

The datasets presented in this study can be found in online repositories. The names of the repository/repositories and accession number(s) can be found below: NCBI SRA PRJNA749474.

## Ethics Statement

The animal study was reviewed and approved by The Ethics Committee of Nanjing Stomatological Hospital, Medical School of Nanjing University.

## Author Contributions

WW, FY, and QH conceived, designed, and coordinated the study. XW, PY, and LF performed the majority of experiments, and analyzed and interpreted the data. SG and FH contributed partially to data acquisition and analysis. WW, FY, and QH contributed reagents/materials/analysis tools. PP contributed to conceptualization and in-depth data interpretation. WH and LZ contributed partially to data analysis. XW, PY, and LF wrote the paper with help from all the authors. All authors contributed to the article and approved the submitted version.

## Funding

This work was supported by the National Natural Scientific Foundation of China (81870767 & 81570978), the Key Project of Science and Technology Department of Jiangsu Province (BL2014018), the Project of Jiangsu Provincial Medical Youth Talent (QNRC2016118), the Preventive Medicine Project of Jiangsu Province (Y2015004), and the Nanjing Medical Science and Technique Development Foundation (ZKX17033 & YKK16162).

## Conflict of Interest

The authors declare that the research was conducted in the absence of any commercial or financial relationships that could be construed as a potential conflict of interest.

## Publisher’s Note

All claims expressed in this article are solely those of the authors and do not necessarily represent those of their affiliated organizations, or those of the publisher, the editors and the reviewers. Any product that may be evaluated in this article, or claim that may be made by its manufacturer, is not guaranteed or endorsed by the publisher.
