# Supplementary material for: Active Smoking Induces Aberrations in Digestive Tract Microbiota of Rats
Source: Front Cell Infect Microbiol. 2021 Nov 29;11:737204. doi: 10.3389/fcimb.2021.737204 (PMC8668415; doi:10.3389/fcimb.2021.737204)
Supplement: Supplementary file 1 [file DataSheet_1.docx]

**Supplementary Material**

**Active smoking induces aberrations in digestive tract microbiota in rats**

Xiang Wang^†^, Pei Ye^†^, Li Fang^†^, Sheng Ge, Fan Huang, Peter J. Polverini, Weiwei Heng, Lichun Zheng, Qingang Hu^*^, Fuhua Yan^*^, Wenmei Wang^*^

**Table of Contents**

**Supplementary Table S1.** Significant difference in relative bacterial abundance in the oral cavity swab samples between the control group and active smoking group.

**Supplementary Table S2.** Significant difference in relative bacterial abundance in the stomach content samples between the control group and active smoking group.

**Supplementary Table S3.** Significant difference in relative bacterial abundance in the small intestine content samples between the control group and active smoking group.

**Supplementary Table S4.** Significant difference in relative bacterial abundance in the cecum content samples between the control group and active smoking group.

**Supplementary Table S5.** Significant difference in relative bacterial abundance in the colon content samples between the control group and active smoking group.

**Supplementary Figure S1.** Analyses show that active smoking decreases body weight gain of rats.

**Supplementary Figure S2.** Matrix heatmap based on Bray-Curtis distance exhibits the overall community structure difference along the digestive tract according to active smoking status.

**Supplementary Figure S3.** Matrix heatmap based on weighted UniFrac distance shows the overall community structure difference along the digestive tract according to active smoking status.

**Supplementary Figure S4.** Effect of active smoking on OTU number along the digestive tract.

**Supplementary video.** The video shows that a rat was actively smoking.

**Supplementary Table S1.** Significant difference in relative bacterial abundance in the oral cavity swab samples according to active smoking status.

| Taxonomic level | Taxonomic name | Control group  Median (IQR) | Active smoking group  Median (IQR) | *p*-value | Alteration trend |
| --- | --- | --- | --- | --- | --- |
| Phylum | Bacteroidetes | 0.1266 (0.0610,1.3540) | 0.0445 (0.0238,0.0616) | 0.004 | ↓ |
|  | Cyanobacteria | 0.0573 (0.0302,0.1386) | 0.2703 (0.1057,0.5311) | 0.027 | ↑ |
| Class | Flavobacteriia | 0.1016 (0.0587,1.3453) | 0.0275 (0.0196,0.0458) | 0.002 | ↓ |
|  | Clostridia | 0.2512 (0.1902,0.5772) | 0.2010 (0.1042,0.2365) | 0.041 |  |
|  | Chloroplast | 0.0573 (0.0285,0.1363) | 0.2703 (0.1045,0.5300) | 0.027 | ↑ |
| Order | Flavobacteriales | 0.1016 (0.0587,1.3453) | 0.0275 (0.0196,0.0458) | 0.002 | ↓ |
|  | Pseudomonadales | 3.4582 (2.0732,7.4260) | 0.8907 (0.3428,2.1926) | 0.011 |  |
|  | Turicibacterales | 0.2820 (0.1648,0.5499) | 0.1206 (0.0641,0.2243) | 0.018 |  |
|  | RF39 | 0.0024 (0.0017,0.0046) | 0.0000 (0,0.0011) | 0.025 |  |
|  | Clostridiales | 0.2512 (0.1902,0.5772) | 0.2010 (0.1042,0.2365) | 0.041 |  |
|  | Bacillales | 0.3798 (0.1560,0.5471) | 0.9005 (0.6416,1.3207) | 0.001 | ↑ |
|  | Streptophyta | 0.0573 (0.0250,0.1363) | 0.2764 (0.1212,0.5779) | 0.018 |  |
|  | Rickettsiales | 0.0096 (0.0017,0.0168) | 0.0341 (0.0091,0.0403) | 0.050 |  |
| Family | Weeksellaceae | 0.1016 (0.0587,1.3453) | 0.0275 (0.0185,0.0458) | 0.002 | ↓ |
|  | Turicibacteraceae | 0.2820 (0.1648,0.5499) | 0.1206 (0.0641,0.2243) | 0.018 |  |
|  | Staphylococcaceae | 0.3549 (0.1471,0.4764) | 0.7836 (0.6071,1.1572) | 0.001 | ↑ |
|  | Planococcaceae | 0.0000 (0,0.0023) | 0.0114 (0.0068,0.0334) | 0.001 |  |
| Genus | *Acinetobacter* | 1.4091 (0.1082,2.7972) | 0.0182 (0.0036,0.0296) | 0.003 | ↓ |
|  | *Elizabethkingia* | 0.0257 (0,1.1987) | 0 (0,0) | 0.008 |  |
|  | *Lactococcus* | 0.0034 (0,0.0046) | 0 (0,0) | 0.014 |  |
|  | *Turicibacter* | 0.2820 (0.1648,0.5499) | 0.1206 (0.0641,0.2243) | 0.018 |  |
|  | *Bacillus* | 0 (0,0) | 0.0046 (0.0034,0.0184) | 0.001 | ↑ |
|  | *Staphylococcus* | 0.3424 (0.1454,0.4674) | 0.7080 (0.4537,0.8686) | 0.003 |  |
|  | *Jeotgalicoccus* | 0.0093 (0.0040,0.0169) | 0.0665 (0.0333,0.0766) | 0.003 |  |
|  | *Sporosarcina* | 0 (0,0) | 0.0047 (0.0023,0.0184) | 0.007 |  |
| Species | *Acinetobacter rhizosphaerae* | 1.1229 (0.1031,2.7713) | 0.0023 (0,0.0047) | 0.001 | ↓ |
|  | *Elizabethkingia meningoseptica* | 0.0257 (0,1.1987) | 0 (0,0) | 0.008 |  |
|  | *Lactococcus garvieae* | 0.0034 (0,0.0046) | 0 (0,0) | 0.014 |  |
|  | *Corynebacterium stationis* | 0.0081 (0.0023,0.0127) | 0.0868 (0.0227,0.1856) | 0.002 | ↑ |
|  | *Bacillus thermoalkalophilus* | 0 (0,0) | 0.0045 (0.0011,0.0115) | 0.003 |  |

IQR: inter-quartile range

**Supplementary Table S2.** Significant difference in relative bacterial abundance in the stomach content samples according to active smoking status.

| Taxonomic level | Taxonomic name | Control group  Median (IQR) | Active smoking group  Median (IQR) | *p*-value | Alteration trend |
| --- | --- | --- | --- | --- | --- |
| Phylum | None |  |  |  |  |
| Class | None |  |  |  |  |
| Order | None |  |  |  |  |
| Family | 0319−6G20 | 0.0000 (0,0.0029) | 0 (0,0) | 0.039 | ↓ |
|  | Mogibacteriaceae | 0.0000 (0,0.0006) | 0.0024 (0.0011,0.0138) | 0.013 | ↑ |
| Genus | *Clostridium* | 0.6409 (0.0200,1.9664) | 0.0161 (0.0092,0.0332) | 0.022 | ↓ |
|  | *Kocuria* | 0 (0,0) | 0.0023 (0,0.0035) | 0.003 | ↑ |
|  | *Desulfovibrio* | 0.0011 (0,0.0029) | 0.0091 (0.0035,0.0274) | 0.009 |  |
|  | *Bilophila* | 0 (0,0) | 0.0024 (0,0.0150) | 0.012 |  |
|  | *Xanthomonas* | 0 (0,0) | 0.0000 (0,0.0079) | 0.022 |  |
| Species | *Clostridium perfringens* | 0.6373 (0.0125,1.9646) | 0.0113 (0,0.0274) | 0.017 | ↓ |
|  | *Kocuria palustris* | 0 (0,0) | 0.0023 (0,0.0035) | 0.003 | ↑ |

IQR: inter-quartile range

**Supplementary Table S3.** Significant difference in relative bacterial abundance in the small intestine content samples according to active smoking status.

| Taxonomic level | Taxonomic name | Control group  Median (IQR) | Active smoking group  Median (IQR) | *p*-value | Alteration trend |
| --- | --- | --- | --- | --- | --- |
| Phylum | None |  |  |  |  |
| Class | Clostridia | 8.1624 (1.6692,16.7226) | 0.8357 (0.4209,2.8336) | 0.022 | ↓ |
| Order | Clostridiales | 8.1624 (1.6692,16.7226) | 0.8357 (0.4209,2.8336) | 0.022 | ↓ |
| Family | Anaeroplasmataceae | 0.0012 (0,0.0085) | 0 (0,0) | 0.018 | ↓ |
|  | Paraprevotellaceae | 0.0186 (0.0018,0.0472) | 0.0000 (0,0.0027) | 0.019 |  |
|  | Tissierellaceae | 0.0000 (0,0.0158) | 0 (0,0) | 0.039 |  |
| Genus | *Anaeroplasma* | 0.0012 (0,0.0085) | 0 (0,0) | 0.018 | ↓ |
|  | *Dehalobacterium* | 0.0024 (0,0.0231) | 0 (0,0) | 0.018 |  |
|  | *Paraprevotella* | 0.0109 (0,0.0428) | 0.0000 (0,0.0027) | 0.031 |  |
|  | *SMB53* | 0.0000 (0,0.0024) | 0 (0,0) | 0.039 |  |
| Species | None |  |  |  |  |

IQR: inter-quartile range

**Supplementary Table S4.** Significant difference in relative bacterial abundance in the cecum content samples according to active smoking status.

| Taxonomic level | Taxonomic name | Control group  Median (IQR) | Active smoking group  Median (IQR) | *p*-value | Alteration trend |
| --- | --- | --- | --- | --- | --- |
| Phylum | Firmicutes | 68.6093 (63.3089,71.1111) | 62.2103 (57.3941,65.3938) | 0.018 | ↓ |
| Class | 4C0d−2 | 0.2402 (0.1917,0.6823) | 0.0865 (0.0435,0.1635) | 0.007 | ↓ |
|  | Clostridia | 65.8491 (61.4529,68.4550) | 60.0341 (56.1512,63.7595) | 0.050 |  |
|  | Actinobacteria | 0.0042 (0,0.0066) | 0.0172 (0.0042,0.0583) | 0.022 | ↑ |
|  | Mollicutes | 0.3694 (0.2955,0.4601) | 0.5339 (0.3724,0.7130) | 0.050 |  |
| Order | Turicibacterales | 0.3034 (0.1924,0.5977) | 0.1476 (0.0385,0.2696) | 0.018 | ↓ |
|  | Clostridiales | 0.2512 (0.1902,0.5772) | 0.2010 (0.1042,0.2365) | 0.050 |  |
|  | Bacillales | 0.0057 (0,0.0152) | 0.0573 (0.0456,0.0761) | 0.001 | ↑ |
|  | Actinomycetales | 0.0042 (0,0.0066) | 0.0172 (0.0042,0.0569) | 0.014 |  |
| Family | Lachnospiraceae | 21.0771 (19.5089,23.8447) | 13.5882 (10.0932,14.9254) | 0.001 | ↓ |
|  | Clostridiaceae | 0.2979 (0.2165,0.4858) | 0.1894 (0.1120,0.2096) | 0.014 |  |
|  | Turicibacteraceae | 0.3034 (0.1924,0.5977) | 0.1476 (0.0385,0.2696) | 0.018 |  |
|  | Alcaligenaceae | 0.0192 (0.0028,0.0644) | 0.0027 (0,0.0085) | 0.018 |  |
|  | Odoribacteraceae | 0.0527 (0.0422,0.0643) | 0.1775 (0.1278,0.2032) | <0.001 | ↑ |
|  | Staphylococcaceae | 0.0028 (0,0.0148) | 0.0511 (0.0401,0.0725) | 0.001 |  |
|  | Rikenellaceae | 0.2706 (0.1933,0.3042) | 0.3539 (0.3106,0.6888) | 0.004 |  |
|  | Veillonellaceae | 0.5573 (0.3785,0.6221) | 0.7376 (0.6054,1.0474) | 0.009 |  |
|  | Corynebacteriaceae | 0 (0,0) | 0.0028 (0,0.0056) | 0.016 |  |
|  | Dehalobacteriaceae | 0.0320 (0.0161,0.0409) | 0.0430 (0.0272,0.0904) | 0.050 |  |
| Genus | *Clostridium* | 0.2379 (0.1602,0.4794) | 0.1237 (0.0950,0.1738) | 0.004 | ↓ |
|  | *Sutterella* | 0.0192 (0.0028,0.0644) | 0.0027 (0,0.0043) | 0.017 |  |
|  | *Turicibacter* | 0.3034 (0.1924,0.5977) | 0.1476 (0.0385,0.2696) | 0.018 |  |
|  | *Morganella* | 0.0014 (0,0.0084) | 0 (0,0) | 0.018 |  |
|  | *Coprococcus* | 3.0281 (1.6721,3.2479) | 1.7570 (1.0579,2.4400) | 0.041 |  |
|  | *Odoribacter* | 0.0192 (0.0098,0.0450) | 0.1399 (0.1012,0.1859) | <0.001 | ↑ |
|  | *Staphylococcus* | 0.0028 (0,0.0140) | 0.0491 (0.0220,0.0668) | 0.001 |  |
|  | *Jeotgalicoccus* | 0 (0,0) | 0.0055 (0.0014,0.0099) | 0.004 |  |
|  | *Desulfovibrio* | 0.4164 (0.2674,0.5414) | 0.7936 (0.5179,1.0656) | 0.011 |  |
|  | *Bilophila* | 0.1607 (0.1179,0.2204) | 0.2524 (0.1871,0.4062) | 0.014 |  |
|  | *Corynebacterium* | 0 (0,0) | 0.0028 (0,0.0056) | 0.016 |  |
|  | *Paraprevotella* | 0.4861 (0.2950,0.9374) | 1.3558 (0.6494,1.5573) | 0.022 |  |
|  | *Dehalobacterium* | 0.0289 (0.0154,0.0380) | 0.0430 (0.0272,0.0904) | 0.050 |  |
| Species | *Clostridium perfringens* | 0.0000 (0,0.0285) | 0 (0,0) | 0.039 | ↓ |
|  | *Desulfovibrio C21 c20* | 0 (0,0) | 0.0027 (0,0.0058) | 0.012 | ↑ |

IQR: inter-quartile range

**Supplementary Table S5.** Significant difference in relative bacterial abundance in the colon content samples according to active smoking status.

| Taxonomic level | Taxonomic name | Control group  Median (IQR) | Active smoking group  Median (IQR) | *p*-value | Alteration trend |
| --- | --- | --- | --- | --- | --- |
| Phylum | Tenericutes | 0.5117 (0.2742,0.6146) | 0.6717 (0.6060,1.1253) | 0.027 | ↑ |
|  | TM7 | 0.0000 (0,0.0028) | 0.0069 (0.0027,0.0109) | 0.046 |  |
| Class | RF3 | 0.0085 (0.0056,0.0100) | 0.0014 (0,0.0076) | 0.026 | ↓ |
|  | Betaproteobacteria | 0.0252 (0.0215,0.1673) | 0.0137 (0.0034,0.0278) | 0.027 |  |
|  | Actinobacteria | 0.0082 (0.0014,0.0086) | 0.0152 (0.0109,0.0428) | 0.012 | ↑ |
|  | Mollicutes | 0.5002 (0.2658,0.6075) | 0.6690 (0.5991,1.1198) | 0.027 |  |
|  | TM7-3 | 0.0000 (0,0.0028) | 0.0069 (0.0027,0.0109) | 0.046 |  |
| Order | ML615J-28 | 0.0085 (0.0056,0.0100) | 0.0014 (0,0.0076) | 0.026 | ↓ |
|  | Turicibacterales | 0.2789 (0.1795,0.4840) | 0.1385 (0.0496,0.1813) | 0.027 |  |
|  | Burkholderiales | 0.0228 (0.0201,0.1673) | 0.0137 (0.0034,0.0278) | 0.034 |  |
|  | Bacillales | 0.0168 (0.0084,0.0254) | 0.0678 (0.0354,0.1016) | 0.002 | ↑ |
|  | Anaeroplasmatales | 0.0428 (0.0126,0.0768) | 0.1188 (0.0634,0.2078) | 0.021 |  |
|  | CW040 | 0.0000 (0,0.0028) | 0.0069 (0.0027,0.0109) | 0.046 |  |
| Family | Lachnospiraceae | 12.9729 (10.4233,22.0016) | 7.5112 (5.3979,11.6844) | 0.012 | ↓ |
|  | Turicibacteraceae | 0.2789 (0.1795,0.4840) | 0.1385 (0.0496,0.1813) | 0.027 |  |
|  | Odoribacteraceae | 0.0143 (0.0029,0.0432) | 0.2720 (0.1405,0.3425) | 0.001 | ↑ |
|  | Staphylococcaceae | 0.0168 (0.0084,0.0254) | 0.0653 (0.0316,0.1014) | 0.002 |  |
|  | Rikenellaceae | 0.2875 (0.1548,0.4499) | 0.5477 (0.4043,0.9598) | 0.016 |  |
|  | Anaeroplasmataceae | 0.0172 (0.0141,0.0275) | 0.1188 (0.0634,0.2078) | 0.021 |  |
|  | Dehalobacteriaceae | 0.0546 (0.0375,0.0768) | 0.1085 (0.0498,0.1552) | 0.021 |  |
|  | Paraprevotellaceae | 1.4864 (0.4740,2.2918) | 2.3795 (1.8202,8.5522) | 0.034 |  |
|  | F16 | 0.0000 (0,0.0028) | 0.0069 (0.0027,0.0109) | 0.046 |  |
| Genus | *Turicibacter* | 0.2789 (0.1795,0.4840) | 0.1385 (0.0496,0.1813) | 0.027 | ↓ |
|  | *Sutterella* | 0.0228 (0.0070,0.1618) | 0.0041 (0,0.0197) | 0.043 |  |
|  | *Odoribacter* | 0.0143 (0.0029,0.0432) | 0.2216 (0.1131,0.2811) | 0.001 | ↑ |
|  | *Staphylococcus* | 0.0142 (0.0070,0.0254) | 0.0626 (0.0316,0.0939) | 0.002 |  |
|  | *Dehalobacterium* | 0.0546 (0.0341,0.0699) | 0.1030 (0.0480,0.1511) | 0.016 |  |
|  | *Anaeroplasma* | 0.0428 (0.0126,0.0768) | 0.1188 (0.0634,0.2078) | 0.021 |  |
|  | *Butyricicoccus* | 0.0517 (0.0330,0.0692) | 0.0789 (0.0544,0.1064) | 0.043 |  |
| Species | *Clostridium perfringens* | 0.0057 (0.0014,0.0184) | 0 (0,0) | 0.006 | ↓ |
|  | *Butyricicoccus pullicaecorum* | 0.0517 (0.0330,0.0692) | 0.0789 (0.0544,0.1064) | 0.043 | ↑ |

IQR: inter-quartile range

**
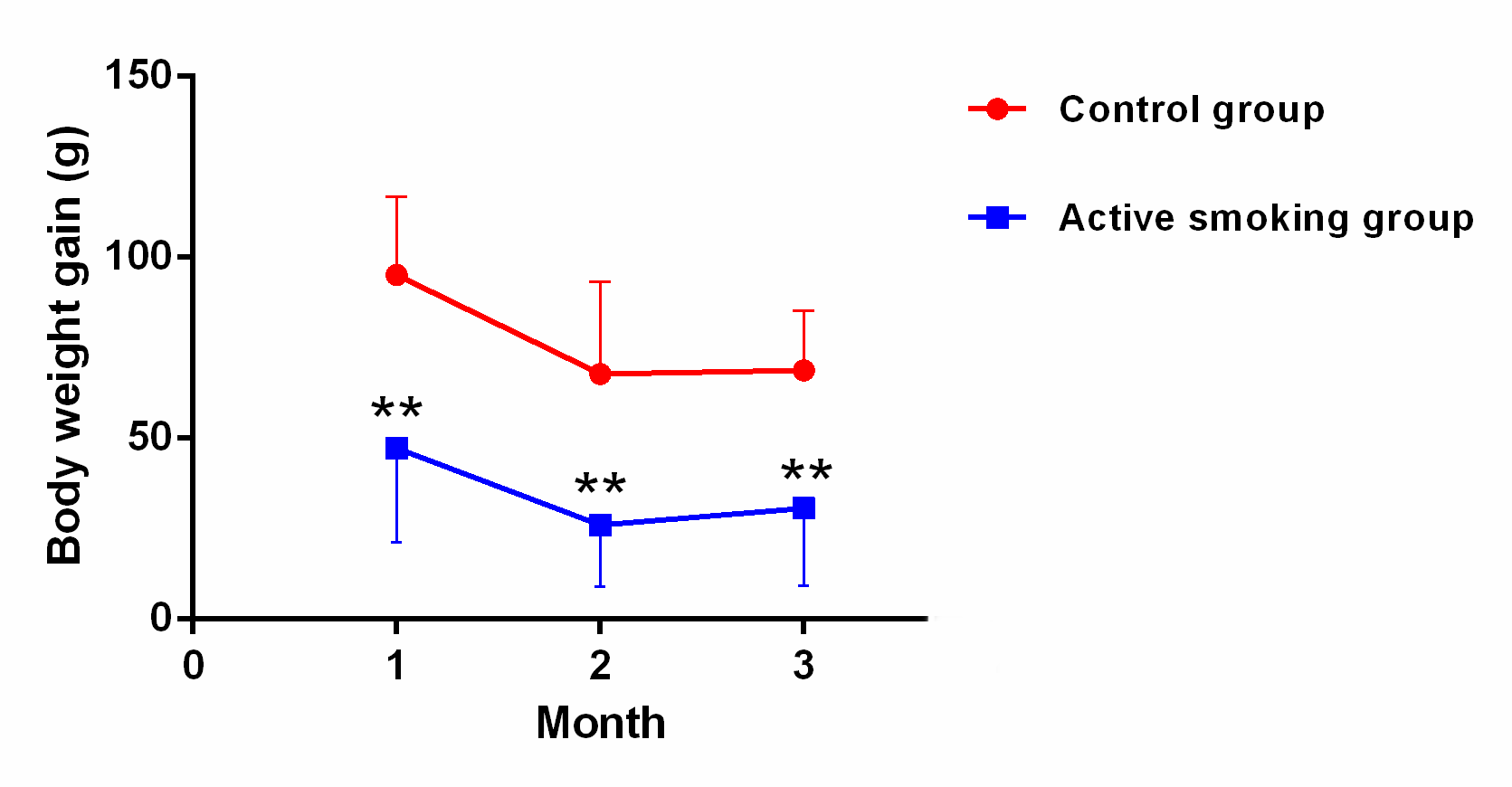
**

**Supplementary Figure S1. Analyses show that active smoking decreases body weight gain of rats.** The weight gain in the active smoking group is significantly lower than that of the control group at various time points. ** denotes *p* < 0.01.

**
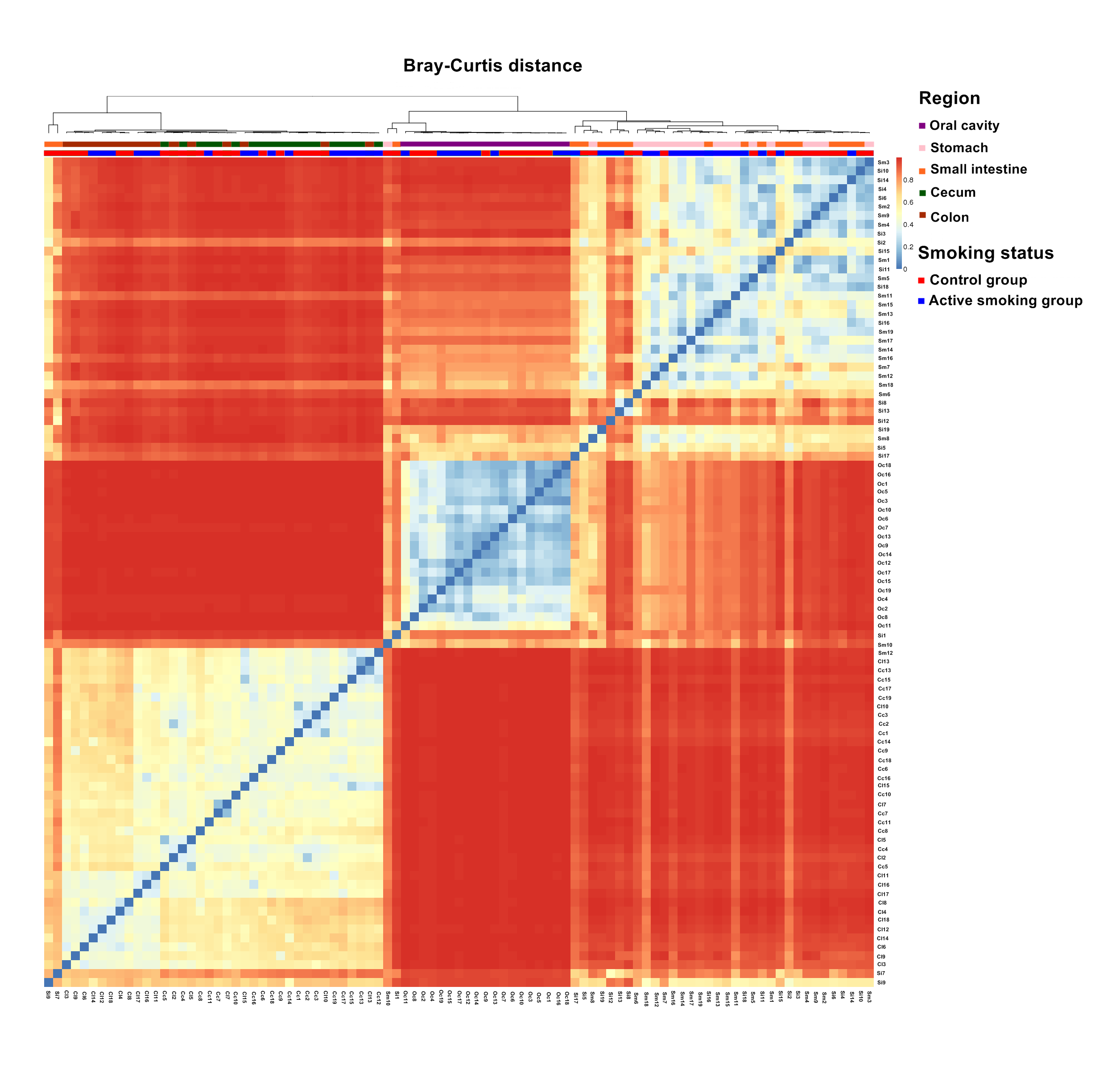
**

**Supplementary Figure S2.** Matrix heatmap based on non-phylogenetic Bray-Curtis distance exhibits the overall community structure difference along the digestive tract according to active smoking status.

**
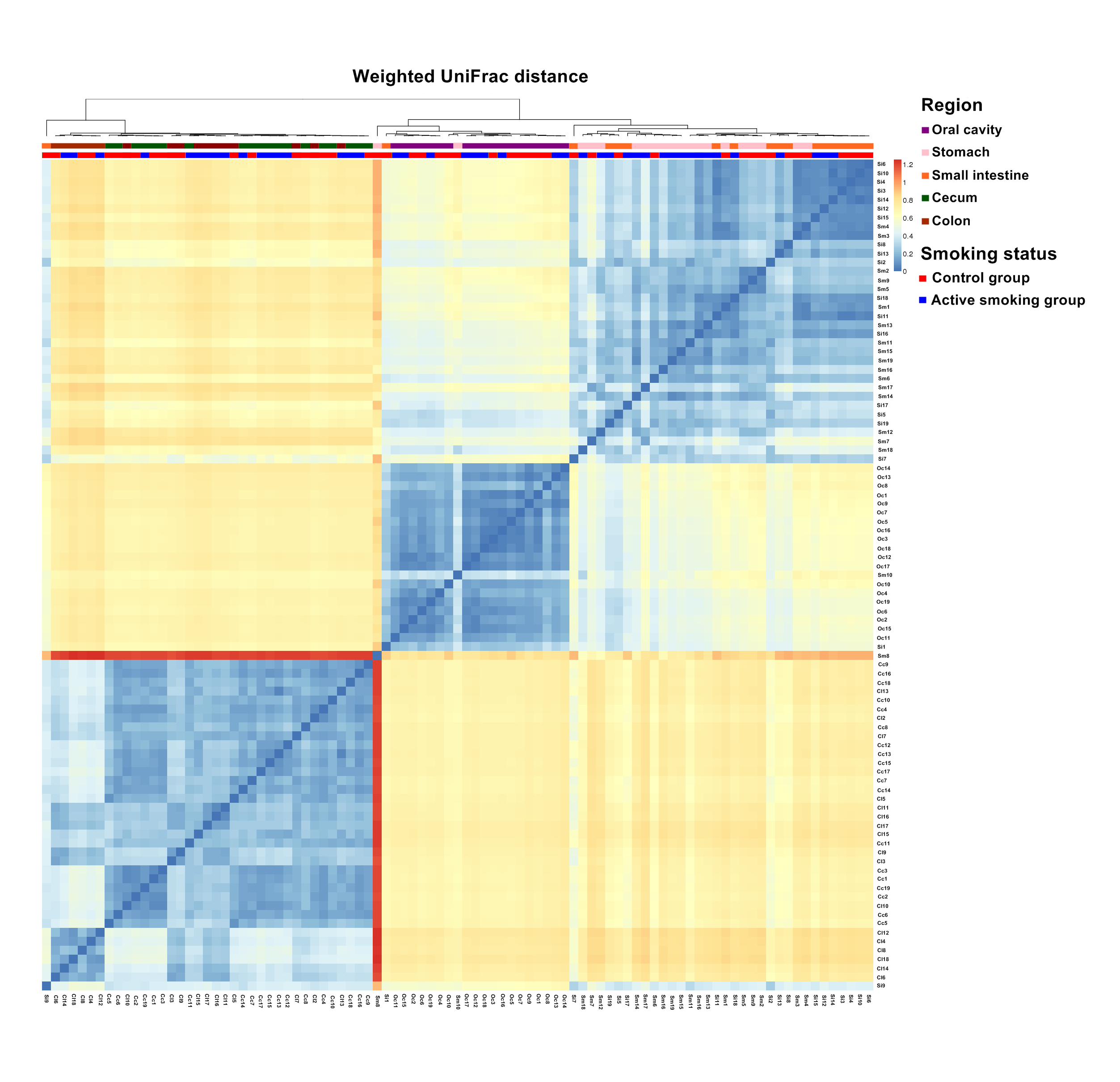
**

**Supplementary Figure S3.** Matrix heatmap based on phylogenetic weighted UniFrac distance shows the overall community structure difference along the digestive tract according to active smoking status.


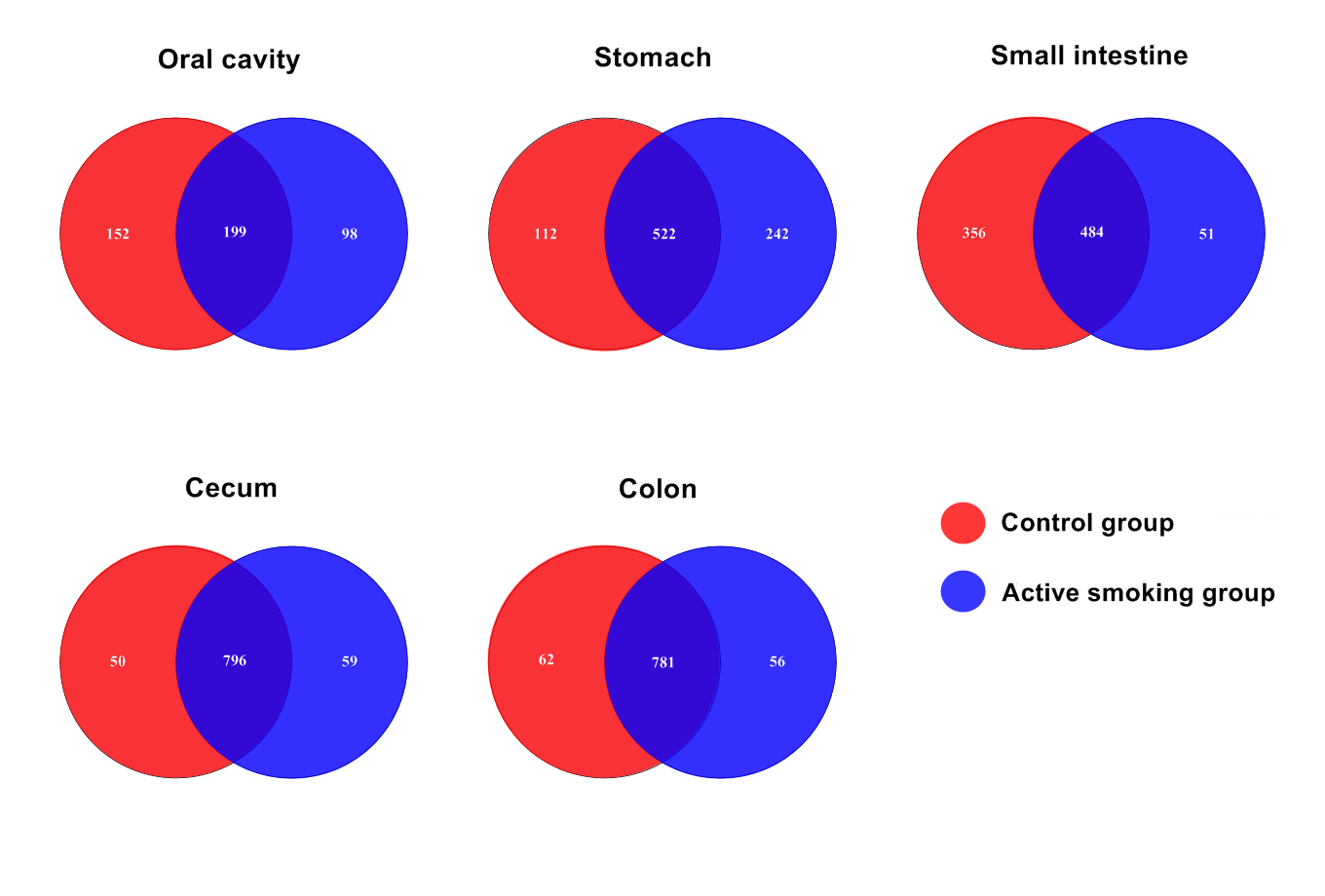
 **Supplementary Figure S4.** Effect of active smoking on OTU number along the digestive tract. Venn diagram showing that fewer unique OTU-annotated bacterial taxa resided in the oral cavity, small intestine, and colon of the active smoking group compared with the control group. In contrast, more unique OTU-annotated bacterial taxa inhabited the stomach and cecum of the active smoking group compared with the control group.
